# ATP Bioluminescence for Assessing the Efficacy of the Manual Cleaning Procedure during the Reprocessing of Reusable Surgical Instruments

**DOI:** 10.3390/healthcare9030352

**Published:** 2021-03-19

**Authors:** Maria Dolores Masia, Marco Dettori, Grazia Maria Deriu, Sabina Bellu, Lisa Arcadu, Antonio Azara, Andrea Piana, Alessandra Palmieri, Antonella Arghittu, Paolo Castiglia

**Affiliations:** 1 Department of Medical, Surgical and Experimental Sciences, University of Sassari, 07100 Sassari, Italy; mdmasia@uniss.it (M.D.M.); lisa.arcadu17@gmail.com (L.A.); azara@uniss.it (A.A.); piana@uniss.it (A.P.); luca@uniss.it (A.P.); castigli@uniss.it (P.C.); 2 University Hospital of Sassari, 07100 Sassari, Italy; mariagrazia.deriu@aousassari.it (G.M.D.); sabina.bellu@aousassari.it (S.B.); arghittu.antonella@gmail.com (A.A.); 3 Department of Biomedical Sciences, University of Sassari, 07100 Sassari, Italy

**Keywords:** healthcare-acquired infections, surgical site infection, reprocessing of surgical instruments

## Abstract

Achieving sterilization by adopting proper practices is essential to ensure that surgical instruments do not transmit microorganisms to patients. As the effectiveness of sterilization mandates effective cleaning, it is necessary to verify the success of cleaning procedures. In this study, we used the adenosine triphosphate (ATP) bioluminescence method for assessing the efficacy of the manual cleaning procedure during the reprocessing of reusable surgical instruments. The ATP bioluminescence assay was performed on 140 surgical instruments of 12 different types, both before being cleaned (baseline) and after each of the cleaning procedures (i.e., decontamination, manual washing, drying, and visual inspection). For each instrument, two swabs were used as follows: one to sample the entire surface (test point 1) and the other to sample the most difficult part of the surface to clean (test point 2). Overall, for each type of instrument, there was a decrease in contamination ranging from 99.6 to >99.9% (log reduction from 2.40 to 3.76). Thus, in order to standardize the assessment of cleanliness, it may be useful to introduce the bioluminescence method into the daily routine or, at least, at regular time intervals as a complementary check combined with visual inspection. This would allow real-time verification of the achievement of an adequate level of cleanliness.

## 1. Introduction

Surgical procedures involve contact between a surgical instrument and a patient’s sterile tissue, mucous membranes, or vascular system. This instrument must be sterile when used as any microbial contamination could result in the introduction of microbes in the surgical site, thus leading to infection. Therefore, achieving sterilization by adopting proper practices is essential to ensure that surgical instruments do not transmit microorganisms to patients. In order to be effective, sterilization must be preceded by thorough cleaning to reduce the bioburden and to remove any foreign material, which interferes with the sterilization process by acting as a barrier to the sterilizing agent [1], from the surfaces of instruments. As the effectiveness of sterilization mandates effective cleaning, it is necessary to verify the success of cleaning procedures. At present, the most commonly used verification method relies on visual inspection, often with the aid of a magnifying glass. This method has several limitations owing to the difficulty of detecting soil on the internal surfaces of the instruments with lumens and, obviously, the subjectivity of the outcome. Some investigators have described the degree of cleanliness of surgical instruments after processing by visual inspection and microscopic examination using a photomicrographic system. On visual inspection, 90.6% of the instruments appeared to be clean; however, microscopic examination revealed residual debris on 84.3% of these [2].

Methods used to verify the cleanliness of reusable surgical instruments during their reprocessing should be easy to perform and objective, and the results should be available in real time in order to allow timely corrective actions [3,4]. During the past few years, data have been published describing the use of various tools and methods to evaluate the effectiveness of environmental surface cleaning; in particular, among these, assays based on the detection of adenosine triphosphate (ATP) through ATP-bioluminescence technology may meet the requirements for ease of use and immediacy of results [5,6,7,8,9,10,11,12,13,14,15,16,17].

ATP is the major energy currency for all living organisms, from the simplest to the most complex, and, therefore, its presence on environmental surfaces indicates the presence of organic matter, including microbiological contamination. Bioluminescence tests are based on the reaction of the enzyme luciferase, extracted from fireflies of the genus *Photinus (Photinus pyralis)*, with its substrate luciferin, as shown in the following equation:

(1) $$D\text{-}\mathrm{Luciferin}+O_{2}+\mathrm{ATP}\overset{\mathrm{Luciferase},\mathrm{Mg}++}{\to }\mathrm{Oxyluciferin}+{\mathrm{CO}}_{2}+\mathrm{AMP}+{\mathrm{PP}}_{i}+hv$$

In the presence of oxygen and magnesium ions, luciferin is oxidized to oxyluciferin and ATP is converted to adenosine monophosphate (AMP) with the release of pyrophosphate (PP_i_) and the emission of light (i.e. bioluminescence). The amount of light emitted in this reaction is directly proportional to the amount of ATP present in the sample [18,19,20].

Owing to its ability to provide real-time results, the ATP bioluminescence method has been widely used since the 1980s in the food industry to monitor the hygienic status of production lines and verify the efficacy of cleaning procedures [6,10,12]. More recently, some investigators have evaluated the application in healthcare settings of ATP bioluminescence assays for monitoring the effectiveness of cleaning procedures for operating theatres, patient rooms, outpatient clinics, and during the reprocessing of medical devices (e.g. endoscopes) [7,11,13,14,16,17,21,22,23,24,25,26,27]. However, to the best of our knowledge, few studies have dealt with the evaluation of the cleanliness of surgical instruments prior to terminal sterilization [3,4,15,28,29].

In this study, we used the ATP bioluminescence method to assess the efficacy of the manual cleaning procedure during the reprocessing of reusable surgical instruments. The aim of the present study was to find an objective technique that could be used (i) to validate the manual cleaning procedure during the reprocessing of reusable surgical instruments; and (ii) to enable, in real time, the material to be sent for terminal.

## 2. Materials and Methods

### 2.1. Bioluminescence Assay

The ATP bioluminescence assay was performed using test devices (3M Clean-Trace ATP System, 3M Italia Srl, Milan, Italy) that consist of specialized swabs for sampling surfaces and ATP bioluminescence reaction cuvettes containing the test enzyme solution (luciferin-luciferase), as well as a hand-held luminometer for detecting and recording the amount of ATP present on swabs. After sampling, each swab was placed into an ATP bioluminescence reaction cuvette and shaken rapidly from side-to-side for at least 5 seconds to mix the sample and the reagent. During this time, the ATP collected reacted with luciferin in the presence of luciferase with the release of energy in the form of light (bioluminescence). The reaction cuvette was then placed in the luminometer, which provided a digital readout of the amount of light, expressed as relative light units (RLUs). The greater the level of ATP collected, the greater the amount of light generated by the test and, therefore, the higher the RLU level produced.

### 2.2. Samples’ Analysis

The study was conducted on freshly used surgical instruments awaiting reprocessing. The structures of these instruments vary widely in complexity and, consequently, some are more difficult to clean than others. In particular, a total of 140 surgical instruments of 12 different types were sampled. According to the personnel charged with reprocessing, 99 were hard to clean and 41 were relatively easy to clean.

All instruments were subjected to analysis immediately after use; prior to manual cleaning (baseline); and after each step of the cleaning procedure: decontamination (step 1), manual washing (step 2), and drying and visual inspection prior to undergoing sterilization (step 3). Every time, for each instrument, two specialized swabs were used as follows: one to sample the entire surface (test point 1) and the other to sample the most difficult part of the surface to clean (test point 2) (Table 1).

At baseline and at each step, the selected instrument was sampled at the same test points.

Before sampling the test instruments, a blank swab (no sample) was run to establish the background RLU level.

### 2.3. Statistical Analysis

Data were entered into Excel (Microsoft Office, Microsoft Corporation, Redmond, WA, USA) and analyzed using the STATA software 16 (StatCorp., Austin, TX, USA) and MedCalc (MedCalc Software Ltd, Ostend, Belgium). Data were summarized with absolute and relative (percentages) frequencies, with mean, standard deviation, and median. Statistical comparison for quantitative variables was performed with the Wilcoxon paired test. A *p*-value less than 0.05 was considered statistically significant.

With regard to both the sampling points (namely, test point 1 and test point 2) of each surgical instrument, the RLU value of the contamination level was detected at different times (i.e., baseline, step 1, step 2, and step 3). At the end of each step, and of the entire cleaning process, the logarithmic reduction (Log R) and the percentage of reduction (∆%) of the contamination were calculated.

## 3. Results

Overall, 140 surgical instruments were sampled twice (test points 1 and 2) at baseline and after each step of the cleaning procedure. A total of 1120 swabs were collected (280 per step).

Table 2 shows, for each type of instrument, the average RLU values of the contamination at both test points before cleaning and immediately after each step, and the percentage of reduction and the logarithmic (log) reduction of the same after each step and at the end of the cleaning procedure.

**Figure 1:**
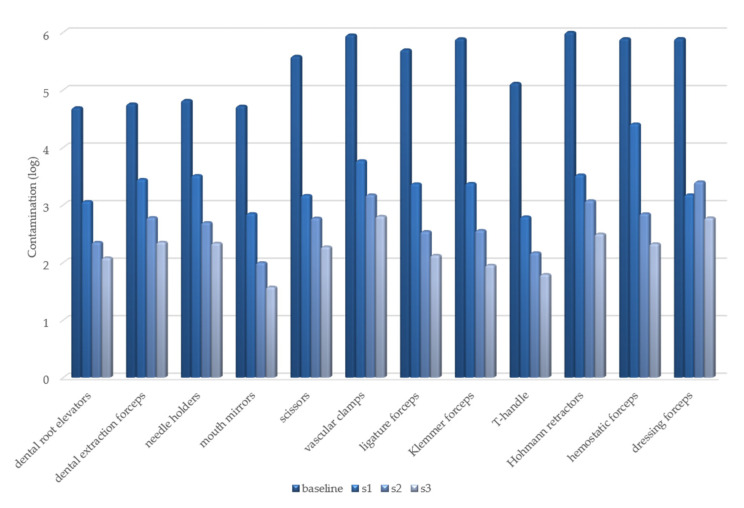
Test point 1: variation in degree of contamination in each cleaning step.

**Figure 2:**
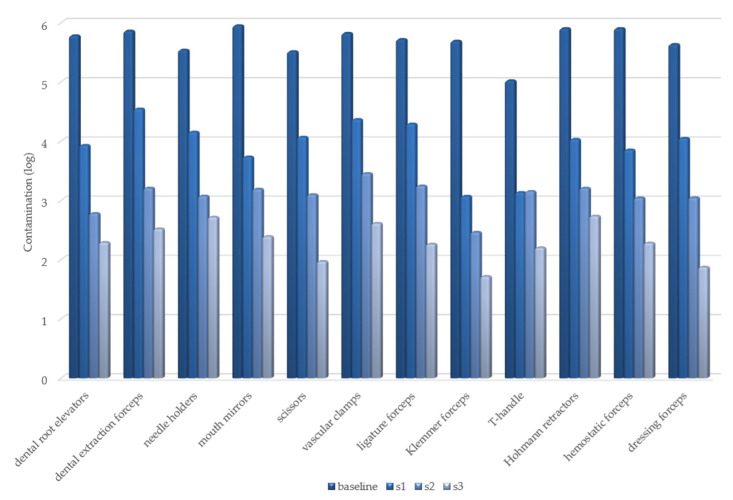
Test point 2: variation in degree of contamination in each cleaning step.

The variation in the degree of contamination, expressed as log-transformed RLU values, is shown in Figure 1 and Figure 2.

Overall, the average values of the contamination of surgical instruments as received from surgery, before being cleaned (baseline), varied widely between different types of instruments, being between 1,004,571.36 and 48,920.65 RLUs (mean 455,767.35, DS ± 376,671.26; median 439,728.56 RLUs) for swabs at test point 1 (tp1), and between 891,602 and 105,037.2 RLU (mean 558,326.36 RLUs, DS ± 232,592.1; median 563,594.31 RLU) for swabs at test point 2 (tp2); the dirtiest instruments were Hohmann retractors, hemostatic forceps, and vascular clamps, while the least contaminated were T-handles. After step 1 (s1), decontamination, RLUs decreased at both sampling points (test points 1 and 2) by more than 95%, ranging from 95.1% to 99.8% depending on the type of instrument, expressing a log reduction ranging from 1.31 to 2.72 log; the lowest efficacy of decontamination was observed in dental extraction forceps and in needle holders at test point 1. A further reduction of the contamination was almost always observed after manual washing (step 2) and after drying (step 3). In some cases, between the sequential steps (after step 1, before step 2 and/or after step 2, before step 3), the RLU values slightly increased rather than decreased. This may have been caused by the handling of surgical instruments and/or by the presence of organic material in the wash water. Comparing, for each type of instrument, the baseline average value of contamination with the average final value (after step 3), there was a decrease in contamination ranging from 99.6 to >99.9% (log reduction from 2.40 to 3.76), without significant differences between the different types of instruments and, for each instrument, between the test points 1 and 2.

## 4. Discussion

The role of external risk factors in the pathogenesis of surgical site infection (SSI), a major complication following surgery and now among the most common and the most costly of all hospital-acquired infections, is well recognized [30,31,32,33]. External risk factors for SSI include inadequate sterilization of surgical instruments [30,33]. Several cases and outbreaks of infections associated with this condition are reported in the literature [30,34].

Furthermore, great attention must also be paid to the risk of transmission of prions through contaminated surgical instruments. Prions are extremely resistant to conventional chemical and physical decontamination and sterilization procedures and have high binding affinity to and tenacity on steel surfaces [35,36,37]. Therefore, specific procedures are mandatory for the reprocessing of non-disposable instruments used on patients with Creutzfeldt–Jakob disease (CJD) or those with a recognizable risk of the same [35,38,39,40]. Such procedures, however, are not commonly practiced for the routine maintenance of surgical instruments used on patients without a recognizable risk of CJD (e.g., during the preclinical phase).

In all surgical procedures, it is of the utmost importance to avoid the transmission of infectious diseases via surgical instruments. When reusable instruments are utilized, to guarantee their sterility, it is necessary to ensure that the sterilization process has been carried out effectively. The sterilization process is considered a “special process”, because its output, product sterility, cannot be verified retrospectively by testing the product before use. The only guarantee of proper sterilization is the scrupulous execution of all steps of the process itself. In particular, it is of crucial importance to adhere to the proper cleaning procedure to have a successful outcome of the sterilization process as the “dirt” that remains on the surfaces of instruments interferes with the sterilization agent, thereby compromising the outcome [1]. “You can’t sterilize dirt” is in fact an axiom often employed to signify the critical importance of the cleaning stage in the reprocessing of reusable devices [4]. Furthermore, accurate routine cleaning of reusable surgical instruments by reducing the presence of dirt could also reduce, in principle, the potential risk of prion transmission.

Currently, the most-frequently used method of assessing cleanliness relies on visual inspection, which has many limitations as it does not enable the detection of dirt on internal surfaces and, obviously, the outcome is subjective and can be influenced by the fatigue level of the person performing the inspection (time spent working, number of instruments, and so on). In a few cases, methods based on microbiological control or on the determination of residues of blood or protein are used. However, these methods have considerable disadvantages, mainly, (i) the results of microbial tests are not immediately available; (ii) some microorganisms require specific growth conditions and a long cultivation period, and thus are missed by routine microbiological methods; and (iii) the interpretation of the results of residual protein tests is highly subjective as it usually requires comparison with a color chart [3,4,15].

In this work, we used an ATP bioluminescence-based method to assay, through the detection of residual dirt, the effectiveness of manual cleaning during the reprocessing of surgical instruments. The measurement of residual dirt, carried out in each step of the cleaning process, quickly showed in all the types of surgical instruments examined, also at their most critical points and despite high initial levels of dirt, a reduction in the initial (baseline) contamination which was higher than 95% already following the first step and a total or almost total reduction at the end of the process. Indeed, if objectively inadequately-cleaned instruments are identified, corrective actions (re-cleaning) can be promptly carried out, thereby avoiding ineffective sterilization and possible damage to patients. Moreover, in addition to objectively validating the cleaning procedure adopted, this system, by virtue of its ability to store data, makes it possible to have documentation certifying the result. This aspect should not be underestimated, firstly because it attests that the checks have been carried out and gives their results, which could be useful in cases of dispute, and secondly because documentation of the outcome could be a useful element in the training programs of the personnel tasked with cleaning surgical instruments. We believe that this system constitutes an effective tool for assessing the quality of the cleaning process preliminary to sterilization. Therefore, in order to standardize the assessment of cleanliness, it may be useful to incorporate the bioluminescence method into daily routine or, at least, to perform it at regular time intervals as a complementary check combined with visual inspection. This would allow real-time verification that an adequate level of cleanliness has been achieved. Such a level of cleanliness is an essential prerequisite for ensuring effective sterilant activity, as requested in the CDC Guidelines for Disinfection and Sterilization in Healthcare Facilities. These guidelines state that, “although the effectiveness of high-level disinfection and sterilization mandates effective cleaning, no “real-time” tests exist that can be employed in a clinical setting to verify cleaning. If such tests were commercially available they could be used to ensure an adequate level of cleaning” [1].

With this in mind, in the absence of benchmark threshold RLU values for surgical instruments after manual cleaning (to the best of our knowledge, there are some indications only for the washer-disinfector step, but not for the manual cleaning step), the method adopted in the absence of automatic washers and often recommended for delicate and/or complex instruments, starting from the average RLU values found at the end of the cleaning process (after step 3) resulting from a reduction in baseline contamination ranging from 99.6 to >99.9%, we propose a pass value <200 RLU. This value expresses the median of residual contamination values found on the clean instruments. 

Finally, some limitations and strengths of the study should be emphasized. In particular, the small variety of surgical instruments evaluated calls for further study to obtain more data on how the method performs even with much more complex instruments. Nevertheless, the reduction in RLUs, which was statistically significant between baseline and S3, suggests the validity of this objective and user-friendly method.

## 5. Conclusions

Adequate sterilization of surgical instruments is a fundamental aspect in the prevention of healthcare-acquired infections. As such, it is essential to ensure that all reprocessing steps are appropriate. Consequently, for sterilization to be effective, it must be preceded by adequate cleaning. 

Our study involved the use of the ATP bioluminescence method to validate the manual cleaning procedure during the reprocessing of reusable surgical instruments. Overall, the method allowed easy, objective, and real-time assessment of the degree of reduction of the contamination level. Therefore, the authors believe that this method may be considered as part of routine reprocessing activities or, at least, on a periodic basis.

## Figures and Tables

**Table 1:** Surgical instruments tested.

Instrument Type	No.	Classification	Test Point	Swabbed Point
Dental root elevators	17	Easy	T1	Entire surface
			T2	Blade
Dental extraction forceps	17	Hard	T1	Entire surface
			T2	Inner beak
Needle holders	15	Hard	T1	Entire surface
			T2	Criss-cross striation
Mouth mirrors	3	Easy	T1	Entire surface
			T2	Mirror
Scissors	13	Hard	T1	Entire surface
			T2	Between blades
Vascular clamps	7	Hard	T1	Entire surface
			T2	Grooves on tip
Ligature forceps	18	Hard	T1	Entire surface
			T2	Grooves on tip
Klemmer forceps	5	Hard	T1	Entire surface
			T2	Grooves on tip
T-handle	10	Easy	T1	Entire surface
			T2	Tip
Hohmann retractors	11	Easy	T1	Entire surface
			T2	Tip
Hemostatic forceps	8	Hard	T1	Entire surface
			T2	Grooves on tip
Dressing forceps	16	Hard	T1	Entire surface
			T2	Grooves on tip

**Table 2:** Results of determination of adenosine triphosphate (ATP) bioluminescence in surgical instruments before cleaning and after each step: average relative light units (RLUs) values; logarithmic reduction (Log R); percentage of reduction (∆%).

Surgical Instruments	Test Point	Baseline RLU	S1 RLU	Log R	∆%	S2 RLU	Log R	∆%	S3 RLU	Log R	∆%	Final Log R	Final ∆%
Dental root elevators	1	48,920.7	1145.1	1.63	97.7 *	225.1	0.71	80.3 *	120.9	0.27	46.3 *	2.61	99.8 *
	2	605,685.3	8414.8	1.86	98.6 *	600.7	1.15	92.9 *	194.3	0.49	67.7 *	3.49	100 *
Dental extraction forceps	1	57,009.2	2772.3	1.31	95.1 *	602.8	0.66	78.3 *	224.8	0.43	62.7 *	2.4	99.6 *
	2	723,971.2	35,042.9	1.32	95.2 *	1614.8	1.34	95.4 *	330.9	0.69	79.5 *	3.34	100 *
Needle holders	1	65,732.7	3246.3	1.31	95.1 *	493.7	0.82	84.8 *	215.8	0.36	56.3 *	2.48	99.7 *
	2	344,719.5	14,261.5	1.38	95.9 *	1184.3	1.08	91.7 *	520.7	0.36	56 *	2.82	99.8 *
Mouth mirrors	1	52,249	706.3	1.87	98.6 †	99	0.85	86 †	37.3	0.42	62.3 †	3.15	99.9 †
	2	891,602	5421	2.22	99.4 †	1547	0.54	71.5 †	245.7	0.8	84.1 †	3.56	100 †
Scissors	1	383,413.5	1463.8	2.42	99.6 *	591.1	0.39	59.6 *	186.7	0.5	68.4 *	3.31	100 *
	2	325,265.1	11,680.3	1.44	96.4 *	1249.5	0.97	89.3 *	93.2	1.13	92.5 *	3.54	100 *
Vascular clamps	1	900,930.3	5865.1	2.19	99.3 *	1492.6	0.59	74.6	633	0.37	57.6	3.15	99.9 *
	2	664,129.1	23,273.3	1.46	96.5 *	2846.6	0.91	87.8 *	410.4	0.84	85.6 *	3.21	99.9 *
Ligature forceps	1	496,043.7	2331	2.33	99.5 *	344.1	0.83	85.2 *	133	0.41	61.3 *	3.57	100 *
	2	521,503.3	19,476.8	1.43	96.3 *	1754.7	1.05	91 *	182.8	0.98	89.6 *	3.46	100 *
Klemmer forceps	1	773,259.6	2365.4	2.51	99.7 †	360.2	0.82	84.8 †	89.6	0.6	75.1 †	3.94	100 †
	2	490,343.4	1.177.8	2.62	99.8 †	289.4	0.61	75.4 †	52	0.75	82 †	3.97	100 †
T-handle	1	130,234.8	620.7	2.32	99.5 *	147.3	0.62	76.3 *	61.8	0.38	58	3.32	100 *
	2	105,037.2	1362.5	1.89	98.7 *	1415.1	−0.02	−3.9	158.6	0.95	88.8 *	2.82	99.8 *
Hohmann retractors	1	1,004,571.4	3326	2.48	99.7 *	1182.3	0.45	64.5 *	312.3	0.58	73.6	3.51	100 *
	2	798,895.8	10,747.8	1.87	98.7 *	1613.3	0.82	85 *	544.1	0.47	66.3	3.17	99.9 *
Hemostatic forceps	1	776,224.3	25570	1.48	96.7 *	703	1.56	97.3	210.9	0.52	70 *	3.57	100 *
	2	798,215.9	7117.6	2.05	99.1 *	1105.5	0.81	84.5 *	190.9	0.76	82.7 *	3.62	100 *
Dressing forceps	1	780,619.2	1500.7	2.72	99.8 *	2518.9	−0.22	−67.9	594.8	0.63	76.4 *	3.12	99.9 *
	2	430,548.6	11,187.6	1.59	97.4 *	1110.5	1	90.1	74.7	1.17	93.3 *	3.76	100 *

* *p*-value < 0.005; † *p*-value not calculated due to the small sample size.

## Data Availability

The data presented in this study are available on reasonable request from the corresponding author.
